# Concurrent Adjacent Merkel Cell Carcinoma and Chronic Lymphocytic Leukemia without Simultaneous Merkel Cell Polyomavirus Detection: A Case Series

**DOI:** 10.3390/dermatopathology8020025

**Published:** 2021-06-07

**Authors:** Rayan Saade, Saleh Najjar, Mustafa Erdem Arslan, Peter Rady, Stephen K. Tyring, Tipu Nazeer

**Affiliations:** 1 Department of Pathology and Laboratory Medicine, Albany Medical Center, Albany, NY 12208, USA; saaderayan@gmail.com (R.S.); najjars@stanford.edu (S.N.); mearslanmd@gmail.com (M.E.A.); 2 Department of Dermatology, McGovern Medical School, University of Texas Health Science Center, Houston, TX 77004, USA; Peter.Rady@uth.tmc.edu (P.R.); stephen.k.tyring@uth.tmc.edu (S.K.T.)

**Keywords:** Merkel cell carcinoma, CLL/SLL, Merkel cell polyomavirus, concurrent tumorigenesis

## Abstract

Background: The association between Merkel cell carcinoma (MCC) and chronic lymphocytic leukemia/small lymphocytic leukemia (CLL/SLL) is well established in the literature. A majority of MCCs are known to be associated with Merkel cell carcinoma polyomavirus (MCPyV), which is postulated to be a possible causative agent linking these two entities. We aim to identify the presence of MCPyV in patients with concurrent adjacent MCC and CLL/SLL. Methods: Archived pathology materials of three cutaneous or surgical excisions with concurrent MCC and CLL/SLL were reviewed. Additional 12-µm sections from paraffin-embedded tissue of these resections were matched with original hematoxylin and eosin-stained slides and used to extract foci from each tumor separately. DNA was extracted from these tissues, and polymerase chain reaction (PCR), utilizing a primer set within a highly conserved “small T” viral DNA region, was done to detect MCPyV. Results: Out of 140 cases of cutaneous or surgical excisions with MCC identified in our electronic medical records (EMR), three had coexisting neighboring CLL/SLL in the same resection specimen. In one case out of three, MCPyV was detected in MCC but not in CLL/SLL. The remaining two cases showed no detection of MCPyV in either MCC or CLL/SLL. Conclusion: MCPyV was not concurrently associated with adjacent MCC and CLL/SLL, indicating that it is not driving simultaneous tumorigenesis, at least in a subset of these cases.

## 1. Introduction

Merkel cell carcinoma (MCC) is a relatively rare primary cutaneous neuroendocrine carcinoma with aggressive clinical behavior. The incidence of MCC has tripled in the last 15 years, making the second most common cause of skin cancer death after melanoma [1,2]. Ultraviolet (UV) light exposure is a well-established risk factor for the development of MCC. More recently, a viral oncogenic pathway involving Merkel cell polyomavirus (MCPyV), a non-enveloped double-stranded DNA polyomavirus, has been described as an alternate mechanism driving malignant transformation [3]. MCPyV DNA was found in up to 80% of MCC cases in several studies [3,4,5]. As such, MCC can either be associated with MCPyV or not, with preliminary studies showing some contradicting results of the significance of this distinction [6,7,8,9].

MCPyV encodes several polyomavirus genes that are responsible for viral genome replication in the host cell, including large T antigen (LTag) and small T antigen (STag) [3]. Integration of the virus and truncating mutation of LTag are the principal genetic events. LTag and Stag drive the cellular proliferation and survival in MCPyV-positive MCC [4,10,11].

The association between MCC and chronic lymphocytic leukemia/small lymphocytic lymphoma (CLL/SLL) is well-documented and might indicate a possible common etiology [12,13]. While a number of factors have been proposed to explain this observation, MCPyV has also emerged as a plausible link between these two entities. Interestingly, MCPyV was also detected in up to 33% of CLL/SLL cases [14,15]. In addition, patients with CLL/SLL have an increased risk of developing MCPyV-positive MCC [16].

To further investigate this association, we aimed to identify the presence of MCPyV in three rare cases of concurrent adjacent MCC and CLL/SLL.

## 2. Materials and Methods

The study was approved by the Institutional Review Board with a waiver of consent. Archived pathology materials from three surgical resections of MCC with concurrent adjacent CLL/SLL, out of 140 cases of MCC excisions identified in our electronic medical records (EMR), were retrieved and reviewed. Hematoxylin and eosin (H&E) and immunohistochemically stained slides from these resections were reviewed by one hematopathologist and two pathology residents for confirmation of diagnosis. Additional 12-µm unstained sections were taken from paraffin-embedded tissue blocks of these resections. Unstained slides were matched with original hematoxylin and eosin (H&E) slides and areas from each tumor were extracted separately using microdissection technique. DNA was extracted from these tissues and polymerase chain reaction (PCR), utilizing a primer set within a highly conserved “small T antigen” viral DNA region, was done to detect MCPyV [17].

### 2.1. Case 1

**Figure 1:**
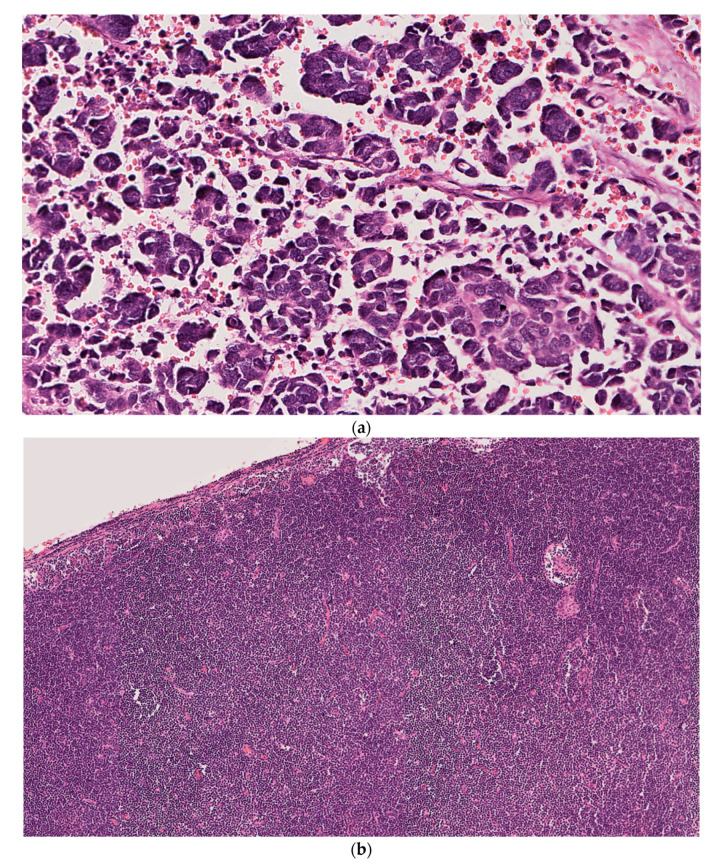

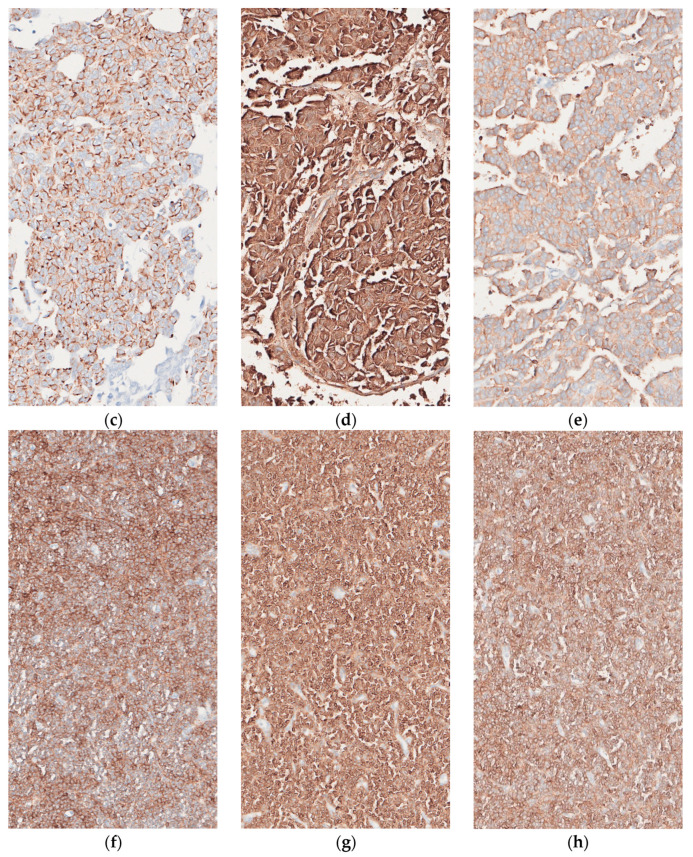
Case 1: (**a**) Left parotid mass showing high-grade neuroendocrine carcinoma composed of small nests and larger aggregates of tumor cells with frequent apoptotic cells and a high mitotic activity. (**b**) Left superior cervical lymph node with abnormal morphology showing diffuse effacement of the normal architecture due to extensive replacement by small, atypical lymphocytes. (H&E; original magnification: (**a**) 20×; (**b**) 7×). Immunohistochemical stains of left parotid mass (**c**–**e**) and left superior cervical lymph node (**f**–**h**). (**c**) CK20 showing dot-like positivity in tumor cells (Magnification 20×). (**d**) Neuron-specific enolase (NSE) diffusely staining tumor cells (Magnification 20×). (**e**). Synaptophysin showing tumor cells with focal membranous positivity (Magnification 20×). (**f**) CD20 diffusely highlights atypical B lymphocytes (Magnification 20×). (**g**) CD5 showing aberrant staining of neoplastic B lymphocytes (Magnification 20×). (**h**) CD23 showing membranous positivity in neoplastic B lymphocytes (Magnification 20×).

A 78-year-old male with a history of CLL/SLL presented with left parotid mass and left-sided cervical lymphadenopathy, underwent left parotidectomy and modified radical neck dissection. A microscopic examination of the parotid gland, para-parotid and cervical lymph nodes showed foci of metastatic undifferentiated carcinoma with neuroendocrine features strongly suggestive of Merkel cell carcinoma on immunohistochemistry (Tumor cells positive for neuron-specific enolase [NSE], synaptophysin [focal], CD56 [focal], MNF-116, AE1/AE3 [focal] and CK20 [dot-like pattern]). Interestingly, the patient had no previous documented history of primary cutaneous MCC or prior surgical resections of skin lesions. More than 90% of the lymph nodes also showed diffuse effacement of architecture and extensive replacement by CLL as demonstrated by immunohistochemistry (atypical lymphocytes positive for CD20, CD5, CD23 and negative for CD3 and cyclin-D1). Detailed clinical history, including lymphoma stage and prior treatment modalities could not be obtained from the available medical records. Tissues used for PCR analysis are shown (Figure 1).

### 2.2. Case 2

**Figure 2:**
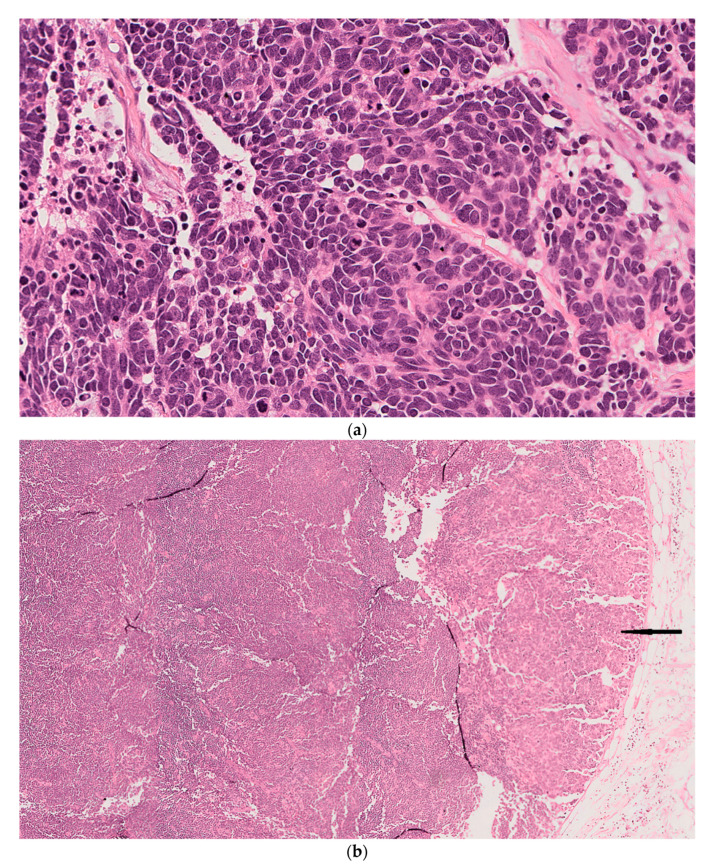

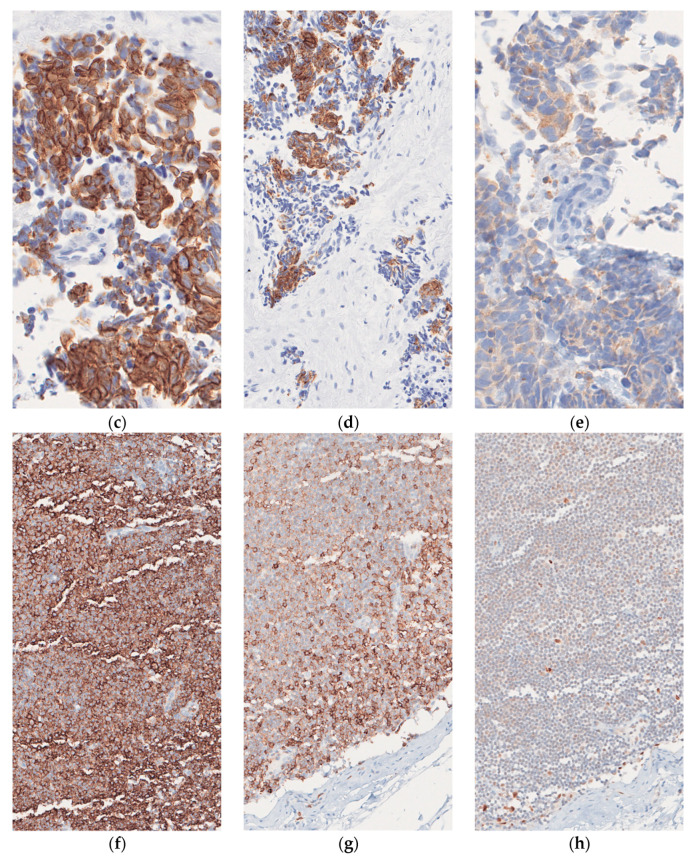
Case 2. (**a**) Left cheek and neck mass showing aggregates and cords of atypical basaloid cells with hyperchromatic nuclei and scant cytoplasm along with numerous mitotic figures and necrotic cells consistent with MCC. (**b**) Left posterior triangle lymph node showing broad effacement of architecture by a dense infiltrate of small monomorphous lymphocytes consistent with CLL. Note the adjacent subcapsular focus of metastatic MCC (Black arrow) (H&E; original magnification: (**a**) 20×; (**b**) 5×). Immunohistochemical stains of left cheek/neck mass (**c**–**e**) and left posterior triangle lymph node (**f**–**h**). (**c**) Low molecular weight keratin showing diffuse cytoplasmic staining of tumor cells (Magnification 40×). (**d**) CK20 showing cytoplasmic and focal dot-like staining in tumor cells (Magnification 20×). (**e**) Synaptophysin showing faint focal positivity in tumor cells (Magnification 40×). (**f**). CD20 diffusely highlights atypical B lymphocytes (Magnification 20×). (**g**) CD5 showing aberrant staining of neoplastic B lymphocytes (Magnification 20×). (**h**) Cyclin-D1 is negative in neoplastic B lymphocytes (Magnification 20×).

This case involves an 85-year-old male with a history of multiple recurrent squamous cell carcinomas (SCC) of the face and scalp, who had previously undergone resection of a large Merkel cell carcinoma involving the left side of his face and the parotid gland 11 months prior to his current presentation followed by re-excision of recurrent disease in the same location five months later. The patient also received adjuvant chemotherapy. He presented with a (3.3 cm) left cheek and neck mass and left posterior triangle lymphadenopathy highly suspicious for recurrent MCC. He underwent radical resection of the mass with left neck node dissection. The final pathology from the cheek/neck resection revealed recurrent MCC and dense monomorphic small lymphocytic infiltrate in the surrounding stroma. Three left posterior triangle lymph nodes also showed replacement by CLL (Stage 1 by modified Rai criteria), the largest of which was also involved by MCC. The diagnosis was confirmed by immunohistochemical testing; Tumor cells from the cheek/neck mass showed positivity for synaptophysin, low molecular weight keratin, CK20 and Ber-EP4 and were negative for TTF-1, chromogranin and high molecular weight keratin. The atypical lymphocytes were positive for CD20, CD5 and CD43 and negative for CD3, cyclin-D1, SOX-11 and CD23. Tissues used for PCR analysis are shown (Figure 2).

### 2.3. Case 3

**Figure 3:**
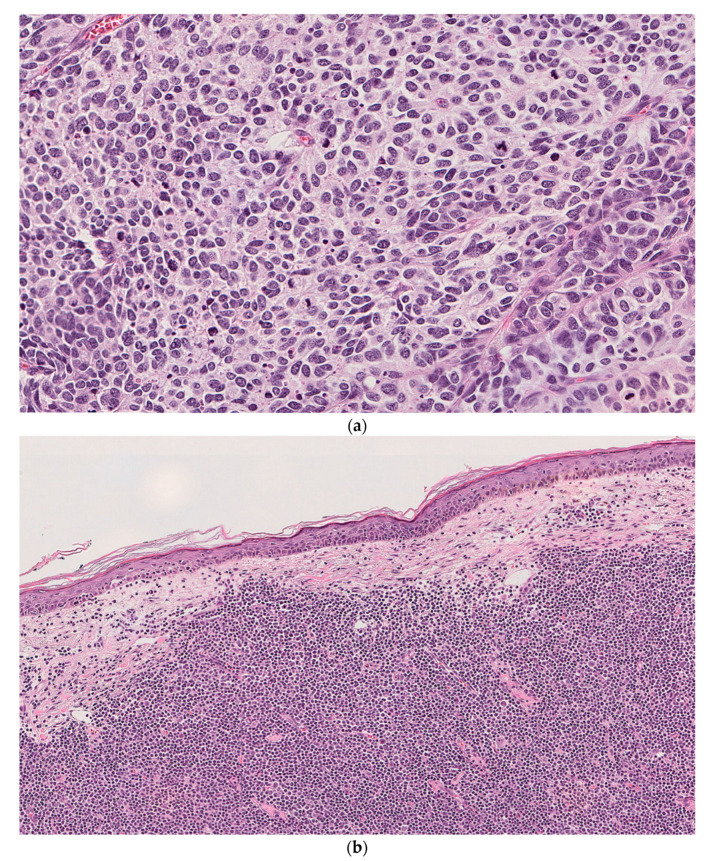

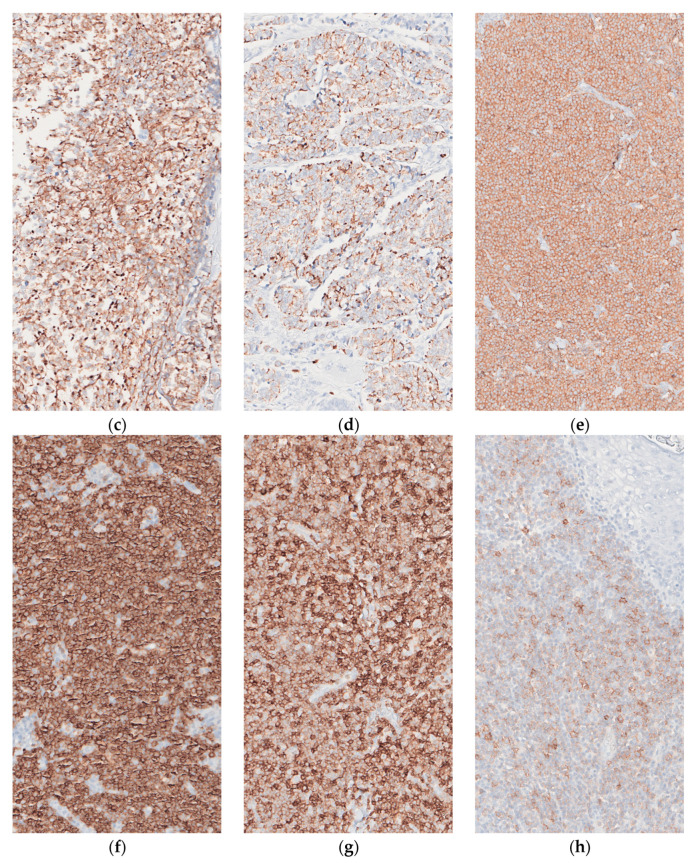
Case 3. (**a**) Right temple mass showing a high-grade neuroendocrine carcinoma with diffuse architecture and focally spindled morphology. (**b**) Sections from the 3 o’clock margin of the right-temple skin excision showing dermal infiltration by an atypical lymphoid proliferation consistent with a low-grade lymphoma further confirmed as CLL/SLL on subsequent immunohistochemistry (H&E; original magnification: (**a**) 20×; (**b**) 10×). Immunohistochemical stains of right temple mass (**c**–**e**) and adjacent right temple skin (**f**–**h**). (**c**) AE1/AE3 showing cytoplasmic and dot-like positivity in tumor cells (Magnification 20×). (**d**) CK20 showing a dot-like staining pattern in tumor cells (Magnification 20×). (**e**) Synaptophysin showing tumor cells with diffuse membranous and cytoplasmic staining (Magnification 20×). (**f**) CD20 diffusely highlights atypical B lymphocytes (Magnification 20×). (**g**) CD5 showing aberrant staining of neoplastic B lymphocytes (Magnification 20×). (**h**) CD23 showing faint membranous positivity in neoplastic B lymphocytes (Magnification 20×).

An 86-year-old male with a history of multiple skin cancers, including basal cell carcinoma (BCC) and SCC status post numerous excisions, presents with a large (8.5 cm) exophytic lesion on the right temple. He also had findings of a right parotid mass on physical examination confirmed on subsequent computed tomography (CT) scan. A biopsy from the skin lesion was consistent with BCC. He underwent a wide local excision of his temporal lesion and right superficial parotidectomy. Microscopic examination of the main skin excision specimen and the right parotid gland showed high-grade neuroendocrine carcinoma consistent with MCC adjacent to and focally colliding with an atypical lymphoid proliferation consistent with CLL/SLL (Stage 1 by modified Rai criteria). Sections from a right facial lymph node also showed a low-grade lymphoma consistent with CLL and involvement by metastatic MCC. Immunohistochemical studies and flow cytometry were performed and supported the diagnosis. Tumor cells from the right temple mass were positive for AE1/AE3, MNF-116, CD56, synaptophysin, CK20 (dot-like pattern) and showed a high proliferation index with Ki67 > 90%. The adjacent atypical lymphocytes were positive for CD19, CD20, CD5, CD23 and showed a low proliferation index as assessed by Ki67. Tissues used for PCR analysis are shown (Figure 3).

## 3. Results

The overall incidence of CLL/SLL in our cohort of MCC cases is 2.1%. In case 1, MCPyV was detected in MCC but not in CLL/SLL. Cases 2 and 3 showed no detection of MCPyV in either MCC or CLL/SLL (Table 1).

## 4. Discussion

MCC can be divided into MCPyV-positive and MCPyV-negative MCC, with some studies showing clinical, morphologic, and behavioral differences between the two “subtypes” [6]. However, separating MCPyV-positive MCC as a distinct entity is not clearly established yet and needs further investigation. MCPyV-positive MCC has a distinct geographical distribution with a higher frequency reported in North America and Europe compared to Australia. This suggests two oncogenic pathways driving malignant transformation in MCC, a viral-induced pathway and an ultraviolet radiation-dependent pathway [18]. In some studies, MCPyV-positive MCC was reported to have a better prognosis compared to MCPyV-negative MCC [6,7]. This could be explained by the fact that MCPyV-negative MCC harbor p53 mutations which, in theory, are associated with poor prognosis and resistance to chemotherapy [19,20]. Even so, other studies did not show any statistically significant difference in prognosis between these two “subtypes”. Morphologically, in-vitro observations showed that MCPyV-positive MCC’s tend to form loose aggregates in culture, also called “classic phenotype” [21], whereas MCPyV-negative MCC’s grow as adherent spindle cells otherwise termed “variant phenotype” [10].

MCPyV encodes several polyomavirus genes, including large T antigen (LTag) and small T antigen (STag), viral proteins 1, 2 and 3 (VP1, VP2, VP3), which are required for viral replication and capsid formation [3]. LTag and STag are primarily responsible for viral genome replication following infection and integration into host DNA. MCPyV-positive MCC’s have a distinct signature mutation that affects the LTag gene. This mutation results in a truncated form of the LTag protein at the C-terminal end, thereby disabling the helicase domain activity [10]. The mutated LTag protein acts as the principal oncoprotein that drives cell proliferation and survival, namely through interaction and inactivation of host cell tumor suppressor genes such as retinoblastoma (Rb) and p53 [4,22]. Experimental knockdown studies of LTag gene showed cell cycle arrest and death of MCPyV-infected MCC cells, supporting the essential role LTag plays as an oncoprotein which in turn makes it an attractive target for the development of molecular targeted therapy [23]. Shuda et al. showed that STag also has an oncogenic effect; It is expressed in most MCC tumors and is required for tumor growth by acting downstream of the mTORC1 signaling pathway [11]

There is a strong association between MCC and CLL/SLL. Patients with CLL have a consistent three- to sevenfold increased risk of developing MCC [13]. Moreover, in a large cohort of 4336 patients from the Finnish Cancer registry and Helsinki University Central Hospital, Koljonen et al. demonstrated a substantially increased risk for MCC following CLL diagnosis and vice-versa. The standardized incidence ratio (SIR) for CLL after the diagnosis of MCC was highly elevated, 17.9 (95% confidence interval (CI), 2.2–64.6; *p* < 0.001), and the SIR for MCC after the diagnosis of CLL was also elevated, 15.7 (3.2–46.0, *p* < 0.01). Importantly, MCPyV DNA was frequently present in MCC’s that occur in CLL patients.16 Nonetheless, while some studies estimated that CLL was 30-fold overrepresented among MCC patients [24], others showed mixed results for the relative risk of lymphoid neoplasia after MCC diagnosis [13].

The association between MCC and CLL/SLL could possibly be attributed to a common etiological agent. In fact, CLL/SLL shares similar demographics with MCC [25]. In addition, MCPyV DNA was found in up to 33% of CLL patients but not follicular lymphoma (FL) patients suggesting either that MCPyV is involved in CLL/SLL pathogenesis or that the immunodeficiency state of CLL/SLL induces low-level MCPyV reactivation [14]. More recently, a novel truncating deletion in the helicase domain of LTag has been identified in a subgroup of MCPyV-positive CLL/SLL patients [15]. Although still controversial, this supports MCPyV as an etiological agent driving the malignant transformation of mature lymphocytes into CLL/SLL, at least in a subset of cases, which further suggests that it could explain the link between MCC and CLL/SLL. MCPyV is indeed lymphotropic [14] and is closely related to the lymphotropic poliomavirus found in African green monkeys, which infects B lymphocytes [26]. In an experimental study done on animals using the simian virus 40 (SV40), a poliomavirus that belongs to the same family as and closely resembles MCPyV, Ter Brugge et al. provided a mouse model for B-CLL through insertion of SV40 large T antigen gene in the immunoglobulin heavy (IgH) locus of mature B cells [27]. On the other hand, Cimino et al. identified MCPyV only in CLL background T cells as opposed to neoplastic B cells [28]. Consequently, the role of MCPyV in the pathogenesis of CLL is still under investigation.

Alternatively, this association can be explained by other factors such as immunosuppression due to both humoral and cell-mediated dysfunction in addition to chemotherapy used to treat CLL, which leads to decreased immune surveillance and subsequently an increased risk of acquiring a secondary malignancy [29]. As a matter of fact, immunosuppression is one of the main risk factors for developing MCC besides older age, UV radiation and Caucasian race [30,31,32,33,34]. Engels found a 13-fold increase in MCC among HIV-positive patients [32], and Miller et al. reported a roughly ten-fold increase after solid organ transplantation [33], further highlighting the importance of immune function in MCC. It is estimated that 10% of MCC patients are immunocompromised [24]. These cases are typically associated with poor survival and a worse prognosis [29].

In our first case, a diagnosis of CLL was made at least six years prior to the patient’s MCC resection. Unfortunately, no history was available pertaining to disease stage and prior chemotherapy treatment or not, although CLL on its own is sufficient to explain the altered immunity which predisposes to MCC. In the remaining two cases, CLL was discovered incidentally at the time of MCC resection, leaving some degree of uncertainty as to the exact timeline of occurrence of each malignancy though, in patient 2, MCC presumptively is more likely to have preceded CLL given his remote diagnosis of advanced MCC for which he received chemotherapy.

MCPyV was detected in MCC but not in CLL of patient 1; in a way, this finding does not support the common viral etiology hypothesis driving the malignant transformation of concomitant CLL and MCC but rather corroborates previous findings demonstrating that CLL is likely a predisposing factor for carcinogenic viral infections such as MCPyV. Patients 2 and 3 had negative MCPyV testing in both MCC and adjacent CLL. Although these observations do not refute the possibility that MCPyV could be involved in partial or simultaneous pathogenesis of MCC and CLL, they do prove that, at least in a subset of cases, the development of concurrent MCC and CLL is not MCPyV-dependent. Morphologically, MCC in patients 2 and 3 showed focally spindled, tight aggregates of tumor cells commensurate with the “variant phenotype” observed in in-vitro studies as compared to the epithelioid configuration and small nested architecture predominating in patient 1.

The cases we present are unique given the proximity of occurrence of MCC and CLL in these resections, which confers a topographic advantage when it comes to validating the idea of two neoplastic processes arising from a common agent, which would likely be detected if present. To our knowledge, this is the first case series examining the presence of MCPyV in concurrent adjacent MCC and CLL. However, given our limited number of cases, further studies are needed, with a larger sample size, which examine the presence and/or expression of MCPyV in concomitant MCC and CLL/SLL and correlates these findings with pertinent clinical variables, namely immunosuppression risk factors and the sequence of development of each malignancy.

## 5. Conclusions

In conclusion, MCPyV is not concurrently associated with adjacent MCC and CLL/SLL, at least in a subset of cases.

## Figures and Tables

**Table 1:** MCPyV detection by PCR in adjacent MCC and CLL/SLL from cutaneous or surgical resections.

	MCPyV in MCC	MCPyV in CLL/SLL
Patient 1	Positive	Negative
Patient 2	Negative	Negative
Patient 3	Negative	Negative
